# Carotid plaque burden across the glycaemic spectrum: a cross-sectional study

**DOI:** 10.1186/s12902-026-02455-z

**Published:** 2026-08-01

**Authors:** İhsan Boyacı

**Affiliations:** Department of Internal Medicine, Faculty of Medicine, Istanbul Medipol University, Molla Gürani Mahallesi, Halıcılar Köşkü Sokak No:11, Fatih, Istanbul, 34093 Türkiye

**Keywords:** Carotid plaque, Prediabetes, Subclinical atherosclerosis, Carotid ultrasonography, Insulin resistance, Type 2 diabetes mellitus

## Abstract

**Background:**

Dysglycaemia accelerates atherosclerosis, but the extent to which carotid plaque burden and morphology differ across the full glycaemic spectrum, and which cardiometabolic factors are most strongly associated with this burden, remain incompletely characterised. This study aimed to evaluate carotid plaque burden and morphology across the glycaemic spectrum, from normoglycaemia to type 2 diabetes mellitus (T2D).

**Methods:**

This cross-sectional study included 169 participants: 24 normoglycaemic controls, 28 with prediabetes, and 117 with T2D stratified by glycaemic control. All underwent clinical, laboratory, and carotid ultrasonographic assessment. Plaque presence, morphology, and stenosis severity were evaluated. Multivariable logistic regression and receiver operating characteristic analyses were performed.

**Results:**

Carotid plaque was present in 50.9% of participants (0% in normoglycaemia, with 8.3% showing isolated intima–media thickness [IMT] increase); a combined outcome of carotid plaque or isolated IMT increase was present in 60.4% overall. Carotid plaque prevalence increased markedly from normoglycaemia (0%) to prediabetes (42.9%) and ranged from 58.6% to 72.4% across the HbA1c-defined T2D subgroups, without a consistent further increase within this range (*p* < 0.001). Plaque morphology also differed across glycaemic categories, with echolucent plaques proportionally more frequent in prediabetes and echogenic plaques predominating in T2D. In the multivariable model, age, urinary albumin-to-creatinine ratio (UACR), and total cholesterol were independent predictors of carotid plaque, whereas diabetes duration, HOMA-IR, and glycated haemoglobin (HbA1c) were not (AUC 0.821).

**Conclusions:**

Carotid plaque burden is markedly higher across the dysglycaemic spectrum than in normoglycaemia, and is already substantial in prediabetes, suggesting early subclinical vascular involvement. Age, UACR, and total cholesterol, rather than HbA1c alone, showed the strongest associations with carotid atherosclerosis. Carotid ultrasonography, including assessment of plaque morphology, may provide additional information for cardiovascular risk stratification beyond glycaemic status alone. This potential role merits evaluation in future prospective studies.

**Graphical Abstract:**

**Figure:**
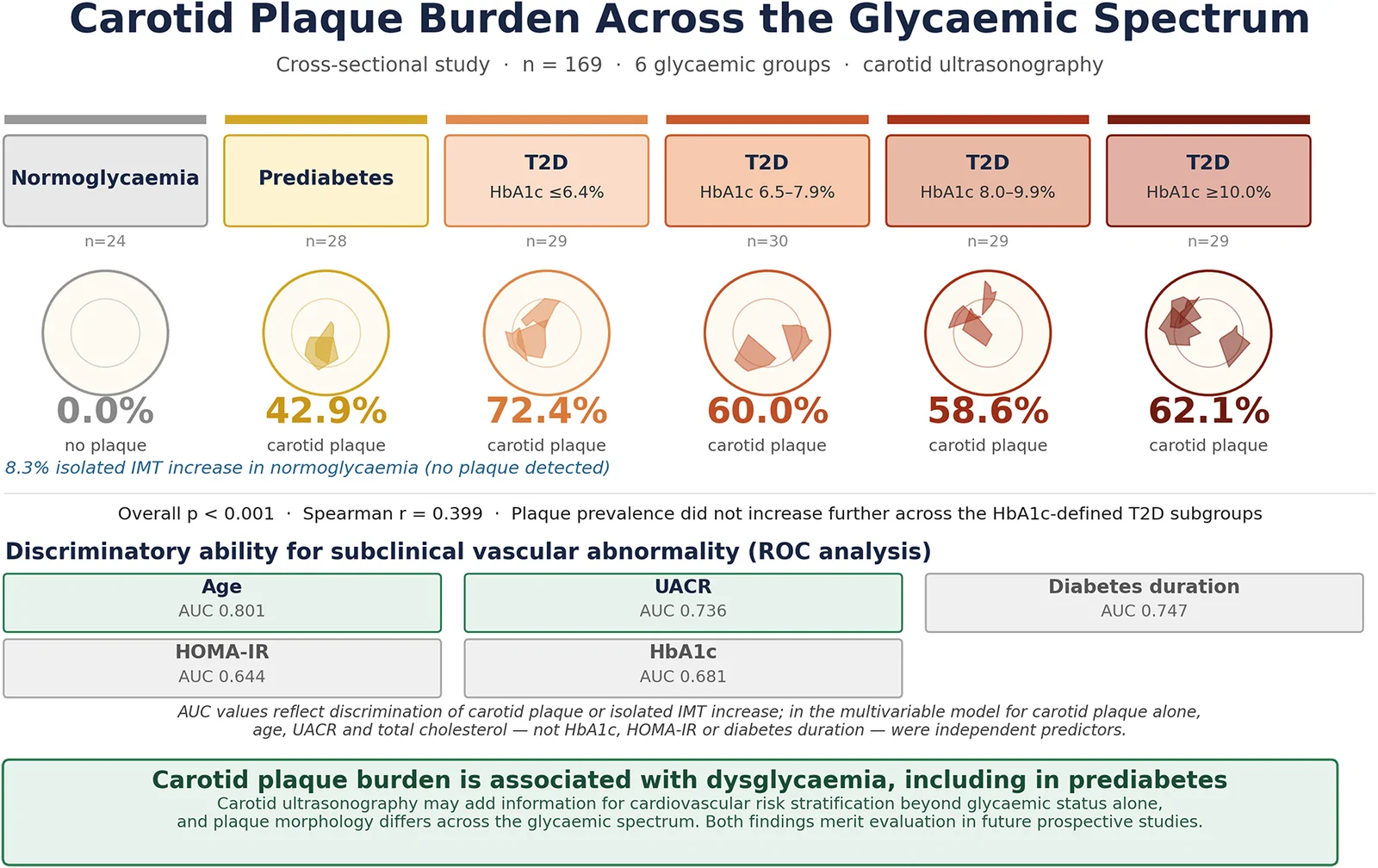

**Supplementary Information:**

The online version contains supplementary material available at https://doi.org/10.1186/s12902-026-02455-z.

## Background

Diabetes mellitus represents one of the most important global health challenges of the 21st century. According to the latest estimates of the International Diabetes Federation, approximately 589 million adults (20–79 years) were living with diabetes worldwide in 2024, with this number projected to increase to approximately 853 million by 2050 [1]. Type 2 diabetes mellitus (T2D) is strongly associated with an increased risk of cardiovascular morbidity and mortality, largely driven by accelerated atherosclerotic processes affecting multiple vascular beds [2]. Consequently, improved identification of early vascular changes has become increasingly important for cardiovascular risk stratification and preventive strategies.

Carotid ultrasonography provides a widely available and non-invasive method for evaluating subclinical atherosclerosis. Although carotid intima–media thickness (IMT) has traditionally been used as an indicator of early vascular alterations, accumulating evidence suggests that the presence of carotid plaque is a more specific marker of atherosclerotic burden and a stronger predictor of future cardiovascular events [3]. In addition to the degree of carotid stenosis, plaque morphology and composition provide complementary information regarding plaque vulnerability and the overall severity of atherosclerotic disease [4, 5].

Prediabetes represents an intermediate metabolic state characterised by insulin resistance, β-cell dysfunction, chronic low-grade inflammation, and early endothelial dysfunction [6, 7]. Beyond chronic hyperglycaemia, vascular injury across the glycaemic continuum is increasingly recognised as a multifactorial process. Small dense LDL particles have been shown to predict subsequent intima–media thickness progression, while platelet hyperreactivity accompanied by a pro-inflammatory profile has also been associated with early vascular damage in prediabetes [8, 9]. Previous studies have reported an increased prevalence of subclinical atherosclerotic plaque in individuals with prediabetes compared with normoglycaemic controls, and have identified age, diabetes duration, and urinary albumin excretion as independent predictors of carotid disease in patients with established T2D [7, 10, 11]. However, these investigations were largely limited to single glycaemic categories and did not evaluate plaque morphology, stenosis severity, or the full spectrum from normoglycaemia to T2D within a single cohort.

Therefore, the present study aimed to evaluate carotid plaque presence, morphology, and stenosis severity across the glycaemic spectrum, ranging from normoglycaemia to varying levels of glycaemic control in T2D. It was hypothesised that carotid atherosclerosis increases in prevalence across glycaemic categories and is more strongly associated with metabolic and renal risk markers than with glycated haemoglobin (HbA1c) alone.

## Methods

### Study design and population

This cross-sectional study was a secondary analysis of a previously established cohort recruited consecutively from the outpatient clinics of a tertiary-care academic center between January and March 2016. Data from this cohort have previously been used to investigate the relationship between the ankle–brachial index and metabolic parameters; however, carotid plaque morphology and stenosis were not analysed in that study. The cohort comprised 169 individuals aged between 40 and 70 years, including normoglycaemic controls, individuals with prediabetes, and patients with T2D. All participants underwent standardised clinical evaluation, anthropometric measurements, laboratory testing, and carotid ultrasonographic assessment. The normoglycaemic control group consisted of individuals attending the Internal Medicine outpatient clinic for routine health evaluation or a general health check-up during the same recruitment period. These individuals had no known history of diabetes or other major chronic disease and were not receiving regular medication. All underwent the same clinical evaluation, physical examination, and laboratory testing as the dysglycaemic participants. The same inclusion and exclusion criteria and assessment protocol were applied to all study groups, and only those fulfilling all predefined eligibility criteria and demonstrating normoglycaemia were assigned to the control group.

Participants were classified according to their glycaemic status based on haemoglobin A1c (HbA1c), fasting plasma glucose, and 75-g oral glucose tolerance test (OGTT) results, using diagnostic thresholds consistent with the American Diabetes Association (ADA) Standards of Care in Diabetes—2025 [12]. Normoglycaemia was defined as HbA1c < 5.7% (39 mmol/mol) with normal OGTT results. Prediabetes was defined as HbA1c 5.7–6.4% (39–47 mmol/mol), impaired fasting glucose (5.6–6.9 mmol/L [100–125 mg/dL]), and/or impaired glucose tolerance (2-hour OGTT glucose 7.8–11.0 mmol/L [140–199 mg/dL]). Participants with established T2D were further stratified according to HbA1c levels and treatment status into four groups: HbA1c ≤ 6.4% (≤ 47 mmol/mol; under ongoing antidiabetic therapy), HbA1c 6.5–7.9% (48–63 mmol/mol), HbA1c 8.0–9.9% (64–85 mmol/mol), and HbA1c ≥ 10.0% (≥ 86 mmol/mol). Participants classified in the HbA1c ≤ 6.4% group had an established prior diagnosis of T2D and were receiving antidiabetic therapy, distinguishing them from individuals with prediabetes, who had no prior diabetes diagnosis and were not receiving glucose-lowering treatment.

Demographic characteristics, medical history including diabetes duration, smoking status, and medication use were recorded for all participants. The original cohort was recruited under ethics approval granted by the Ethics Committee of the Faculty of Medicine, Istanbul Medipol University (Approval No. 10840098-604.01.01-1041; 21 January 2016). The present manuscript represents a retrospective secondary analysis of carotid ultrasound and biochemical data collected from this previously established cohort. Because the present research question was not included in the original study protocol, separate ethics approval for this secondary analysis was obtained from the same Ethics Committee (Approval No: E-10840098-50.04-2669; 13 April 2026), and written informed consent was obtained from all participants at the time of original enrollment, covering the use of their clinical data and biological samples for research purposes. The study was conducted in accordance with the principles of the Declaration of Helsinki.

### Inclusion criteria

Participants aged between 40 and 70 years who underwent comprehensive metabolic evaluation, including HbA1c measurement, fasting plasma glucose testing, OGTT, and carotid ultrasonographic examination were eligible for inclusion. Individuals were required to meet the ADA diagnostic criteria for normoglycaemia, prediabetes, or T2D and to have complete clinical, laboratory, and imaging data available for analysis.

### Exclusion criteria

Participants were excluded if they had a history of malignancy or ongoing cancer treatment, chronic liver disease including cirrhosis, severe malnutrition, anemia or hemolytic disorders, pregnancy, or use of immunosuppressive or antioxidant medications. Individuals with incomplete clinical, laboratory, or carotid ultrasonography data were also excluded from the analysis. All participants included in the final analysis had complete data for the primary model variables following data verification.

### Clinical and laboratory measurements

Anthropometric measurements were performed by trained personnel using standardised procedures. Body weight was measured to the nearest 0.1 kg with participants wearing light clothing and no shoes, and height was measured to the nearest 0.1 cm using a stadiometer. Body mass index (BMI) was calculated as weight in kilograms divided by the square of height in meters (kg/m²). Waist circumference was measured at the midpoint between the lowest rib and the iliac crest, and hip circumference was measured at the level of the greater trochanters according to World Health Organization recommendations [13]. Waist-to-hip ratio (WHR) was subsequently calculated. In addition to conventional anthropometric indices, Body Roundness Index (BRI) was calculated using a validated formula based on waist circumference and height [14].

After an overnight fast of at least 8 h, venous blood samples were collected and analysed in the hospital central laboratory. Fasting plasma glucose (FPG), lipid parameters, creatinine, and high-sensitivity C-reactive protein (hs-CRP) were measured using an automated chemistry analyzer (VITROS 350 Chemistry System, Ortho Clinical Diagnostics, Rochester, NY, USA). Plasma glucose concentrations were determined using an enzymatic method and expressed primarily in SI units (mmol/L), with conventional units provided in parentheses.

HbA1c was measured using a boronate affinity assay (Quo-Lab HbA1c Analyzer, EKF Diagnostics, Cardiff, UK) standardised according to the National Glycohemoglobin Standardization Program (NGSP). Fasting insulin concentrations were determined using electrochemiluminescence immunoassay (Cobas e411 analyzer, Roche Diagnostics, Mannheim, Germany). Insulin resistance was estimated using the homeostasis model assessment for insulin resistance (HOMA-IR), calculated as:

$$\eqalign{ & \:{\text{HOMA - IR}}\:{\text{ = }} \cr & \:[{\mathrm{fasting}}\:{\mathrm{insulin}}\:(\mu \:{\mathrm{U/mL}})\: \times \:\:{\mathrm{fasting}}\:{\mathrm{glucose}}\:({\mathrm{mmol/L}})]\cr &\:{\mathrm{/}}\:{\mathrm{22}}.{\mathrm{5}} \cr} $$

Serum lipid parameters including total cholesterol, low-density lipoprotein cholesterol (LDL-C), high-density lipoprotein cholesterol (HDL-C), and triglycerides (TG) were measured using enzymatic colorimetric assays. The triglyceride-to-HDL cholesterol ratio (TG/HDL ratio) was calculated as an indicator of atherogenic dyslipidemia.

Urinary albumin excretion was assessed from early-morning spot urine samples using an immunoturbidimetric assay (i-CHROMA II analyzer, Boditech Med Inc., Chuncheon, Korea) and expressed as the albumin-to-creatinine ratio (mg/g creatinine). Estimated glomerular filtration rate (eGFR) was calculated using the Chronic Kidney Disease Epidemiology Collaboration (CKD-EPI) equation based on serum creatinine levels.

Blood pressure was measured in the seated position after a period of rest, in both arms, and the mean of the two measurements was used for analysis. Hypertension was defined as systolic blood pressure ≥ 140 mmHg, diastolic blood pressure ≥ 90 mmHg, or current use of antihypertensive medication [15]. Medication use, including statins, antiplatelet agents, renin-angiotensin-aldosterone system (RAAS) blockers (angiotensin-converting enzyme inhibitors or angiotensin receptor blockers), oral antidiabetic agents, and insulin therapy (basal and/or bolus), was recorded from medical records for all participants. Macrovascular disease was defined as the presence of ischaemic heart disease and/or peripheral arterial disease as recorded in the original cohort assessment. Peripheral arterial disease was identified on the basis of an abnormal ankle–brachial index (< 0.90, or > 1.30 indicating medial calcification) rather than a documented clinical event, and therefore largely reflects screen-detected rather than symptomatic disease, whereas ischaemic heart disease was documented in 17 participants (10.1%). Carotid atherosclerosis was excluded from this definition, as it overlaps with the primary study outcome and its inclusion as a covariate would introduce circularity into the multivariable model.

All laboratory analyses were performed according to internal and external quality control procedures. During data verification, a small number of biologically implausible values (one high-density lipoprotein cholesterol value, one waist circumference value, and two low-density lipoprotein cholesterol values recorded as zero) were identified and treated as missing, and were excluded from the corresponding analyses.

### Carotid ultrasound assessment

Carotid artery evaluation was performed using B-mode and Doppler ultrasonography by a single experienced radiologist using a standardised institutional protocol. Examinations were conducted with a high-resolution ultrasound system equipped with a linear-array transducer (Logiq P6 Pro; GE Healthcare, Milwaukee, WI, USA) operating at a frequency of at least 7 MHz.

Participants were examined in the supine position after a resting period. Both right and left common carotid arteries, carotid bulbs, and internal carotid arteries were systematically assessed in longitudinal and transverse planes. IMT and the presence of atherosclerotic plaques were evaluated according to established ultrasonographic criteria.

A carotid plaque was defined, in accordance with the Mannheim Carotid Intima–Media Thickness and Plaque Consensus [16], as a focal structure encroaching into the arterial lumen with a thickness ≥ 1.5 mm or ≥ 50% greater than the surrounding intima–media thickness. Plaque morphology was classified according to the Gray–Weale classification into four types based on echogenicity: type I (uniformly echolucent plaque), type II (predominantly echolucent plaque with small echogenic areas), type III (predominantly echogenic plaque with small echolucent areas), and type IV (uniformly echogenic plaque) [17]. Isolated intima–media thickness increase was defined as a common carotid intima–media thickness ≥ 1.0 mm in the absence of any focal structure meeting the plaque criteria.

Carotid stenosis was graded according to the institutional Doppler ultrasound protocol based on the European Carotid Surgery Trial (ECST) methodology [18, 19]. Doppler velocity measurements—including peak systolic velocity (PSV) and end-diastolic velocity (EDV)—were recorded for each vessel segment at every examination, from which the internal carotid artery-to-common carotid artery (ICA/CCA) PSV ratio could be derived. These measurements were interpreted together with grayscale (B-mode) plaque morphology and luminal narrowing. Because stenosis grading followed this integrated ECST-based clinical assessment rather than a fixed velocity-only algorithm, no single Doppler velocity threshold was used in isolation to define stenosis severity, consistent with multiparametric Doppler assessment approaches recommended by international consensus [20]. When plaque or stenosis was present bilaterally, the carotid artery demonstrating the highest degree of stenosis (maximum percentage stenosis) was selected for participant-level analyses. Accordingly, plaque morphology, stenosis category, and percentage stenosis were recorded on a participant basis for statistical analyses.

### Statistical analysis

Because the present study was a retrospective secondary analysis of an existing cohort, an a priori sample size calculation was not performed. All eligible participants with complete clinical, biochemical, and carotid ultrasonography data were included. A post hoc sample adequacy assessment was therefore performed for the primary outcome (carotid plaque presence across the six glycaemic groups). The observed chi-square effect size was Cohen’s w = 0.449. With a sample size of 169 participants and α = 0.05, the minimum detectable effect size with 80% power was w = 0.276, indicating that the available sample size was adequate to detect a moderate group effect. No missing data were present for any of the model covariates following data verification; accordingly, all analyses, including the multivariable models, were performed in all 169 participants.

Statistical analyses were performed according to a predefined analysis plan. Continuous variables were tested for normality using the Kolmogorov–Smirnov test and visual inspection of histograms. Normally distributed variables were expressed as mean ± standard deviation, whereas non-normally distributed variables were presented as median (interquartile range). Categorical variables were summarised as frequencies and percentages.

Comparisons between two groups were performed using the independent samples t-test or the Mann–Whitney U test, as appropriate. For comparisons across multiple glycaemic groups, the Kruskal–Wallis test was used, followed by post hoc pairwise comparisons when appropriate. Trends across ordered glycaemic categories were evaluated using the linear-by-linear association test and Spearman rank correlation.

Categorical variables were compared using the chi-square test, or Fisher’s exact test when expected cell counts were fewer than five, including the pairwise comparison of each glycaemic group with normoglycaemia. Spearman correlation analysis was used to evaluate the associations between carotid stenosis severity and clinical or biochemical parameters. Urinary albumin-to-creatinine ratio (UACR) was analysed as a continuous variable in all correlation, ROC, and multivariable logistic regression analyses.

Multivariable logistic regression analysis was performed to identify independent predictors of carotid plaque presence. The expanded model included age, diabetes duration, HOMA-IR, UACR, HbA1c, hypertension, total cholesterol, statin use, RAAS blocker use, antiplatelet therapy, insulin therapy, eGFR, macrovascular disease, and oral antidiabetic therapy. These covariates were specified a priori on the basis of clinical relevance and of the variables requested during peer review, rather than by data-driven selection. With 86 events and 14 covariates, the events-per-variable ratio was approximately 6; the expanded model was therefore regarded as an adjustment model rather than a parsimonious prediction model, and individual coefficient estimates are interpreted with corresponding caution. Two sensitivity analyses were performed: one excluding insulin-treated participants (Supplementary Table 3) and one excluding macrovascular disease from the covariate set (Supplementary Table 4).

Receiver operating characteristic (ROC) curve analysis was used to assess the discriminatory ability of selected variables, and the area under the curve (AUC) was calculated using the non-parametric (Mann–Whitney) method, with 95% confidence intervals derived by bootstrap resampling (2000 iterations). Optimal cut-off values were determined using the Youden index. ROC analyses were considered exploratory.

ROC analysis was performed using a combined outcome (carotid plaque or isolated IMT increase) to capture the full spectrum of subclinical vascular abnormalities, whereas multivariable logistic regression was based on carotid plaque presence alone as the primary endpoint. A two-tailed p value < 0.05 was considered statistically significant. All statistical analyses were performed using SPSS software (version 22.0; IBM Corp., Armonk, NY, USA).

## Results

A total of 169 participants were included in the final analysis, including 24 normoglycaemic controls, 28 individuals with prediabetes, and 117 patients with T2D. Among patients with T2D, 29 had HbA1c ≤ 6.4% (≤ 47 mmol/mol), 30 had HbA1c 6.5–7.9% (48–63 mmol/mol), 29 had HbA1c 8.0–9.9% (64–85 mmol/mol), and 29 had HbA1c ≥ 10.0% (≥ 86 mmol/mol).

A combined outcome of carotid plaque or isolated intima–media thickness increase was detected in 102 participants (60.4%), of whom 86 (50.9%) had definite carotid plaque. Baseline demographic, clinical, anthropometric, and laboratory characteristics according to glycaemic groups are presented in Table 1. Detailed baseline characteristics stratified by all six glycaemic subgroups are presented in Supplementary Table 1.

**Table 1:** Baseline characteristics of the study population according to glycaemic group

Variable	Normoglycaemia (*n* = 24)	Prediabetes (*n* = 28)	T2D (*n* = 117)	*p* value
Age (years)	47.96 ± 5.75	52.14 ± 8.64	56.88 ± 8.78	< 0.001
Male sex, n (%)	13 (54.2)	15 (53.6)	60 (51.3)	0.953
BMI (kg/m²)	27.25 ± 3.11	31.79 ± 5.70	34.28 ± 5.56	< 0.001
WHR	0.91 ± 0.06	0.98 ± 0.07	0.99 ± 0.06	< 0.001
BRI	5.07 ± 1.17	7.17 ± 2.25	8.17 ± 2.40	< 0.001
SBP (mmHg)	113.02 ± 7.97	120.62 ± 7.16	131.71 ± 20.15	< 0.001
DBP (mmHg)	78.75 ± 4.72	81.25 ± 2.93	83.95 ± 7.34	< 0.001
Hypertension, n (%)	0 (0.0)	7 (25.0)	81 (69.2)	< 0.001
FPG (mmol/L)	4.7 (4.5–4.9)	5.8 (5.4–6.0)	8.3 (6.9–11.9)	< 0.001
PPG (mmol/L)	4.94 ± 0.58	6.85 ± 1.64	13.03 ± 5.36	< 0.001
HbA1c (%)	5.10 ± 0.18	5.74 ± 0.24	8.26 ± 2.18	< 0.001
Fasting insulin (µU/mL)	6.8 (5.5–9.1)	13.7 (9.1–16.8)	13.0 (8.8–20.2)	< 0.001
HOMA-IR	1.4 (1.2–1.9)	3.4 (2.2–4.4)	5.7 (3.2–8.0)	< 0.001
Total cholesterol (mmol/L)	4.58 ± 1.07	4.94 ± 0.96	5.14 ± 1.13	0.079
LDL-C (mmol/L)	2.53 ± 0.93	2.91 ± 0.91	3.03 ± 0.94	0.045
HDL-C (mmol/L)	1.32 ± 0.36	1.10 ± 0.25	1.08 ± 0.25	0.015
TG (mmol/L)	1.2 (0.8–1.7)	1.9 (1.2–2.3)	2.0 (1.4–3.0)	< 0.001
TG/HDL ratio	2.0 (1.3–3.8)	3.8 (2.4–4.9)	4.4 (2.9–7.2)	< 0.001
UACR (mg/g)	5.0 (3.9–6.2)	7.0 (4.0–8.6)	16.8 (8.6–48.7)	< 0.001
eGFR (mL/min/1.73 m²)	104.87 ± 18.42	94.99 ± 20.51	106.29 ± 24.41	0.156
hs-CRP (mg/L)	1.2 (0.8–1.7)	2.5 (1.4–3.4)	2.9 (2.5–5.8)	< 0.001
Statin use, n (%)	0 (0.0)	1 (3.6)	24 (20.5)	0.015
RAAS blocker use, n (%)	0 (0.0)	3 (10.7)	62 (53.0)	< 0.001
Antiplatelet use, n (%)	0 (0.0)	2 (7.1)	27 (23.1)	0.015
Insulin use, n (%)	0 (0.0)	0 (0.0)	25 (21.4)	0.004
Oral antidiabetic use, n (%)	0 (0.0)	0 (0.0)	96 (82.1)	< 0.001
Macrovascular disease, n (%)	0 (0.0)	19 (67.9)	96 (82.1)	< 0.001

Data are presented as mean ± standard deviation or median (interquartile range) for continuous variables, and as number (percentage) for categorical variables. p values were derived using the Kruskal–Wallis test for continuous variables and the chi-square test for categorical variables. Hypertension was defined as systolic blood pressure ≥ 140 mmHg, diastolic blood pressure ≥ 90 mmHg, or current use of antihypertensive medication. Macrovascular disease was defined as ischaemic heart disease and/or peripheral arterial disease recorded in the original cohort assessment; peripheral arterial disease was ascertained by an abnormal ankle–brachial index rather than a documented clinical event, and carotid atherosclerosis was excluded from this definition. Biologically implausible values identified during data verification (one HDL-C, one waist circumference, two LDL-C values recorded as zero) were treated as missing. BMI: body mass index; BRI: Body Roundness Index; DBP: diastolic blood pressure; eGFR: estimated glomerular filtration rate; FPG: fasting plasma glucose; HbA1c: glycated haemoglobin; HDL-C: high-density lipoprotein cholesterol; HOMA-IR: homeostasis model assessment of insulin resistance; hs-CRP: high-sensitivity C-reactive protein; LDL-C: low-density lipoprotein cholesterol; PPG: postprandial glucose; RAAS: renin-angiotensin-aldosterone system; SBP: systolic blood pressure; T2D: type 2 diabetes mellitus; TG: triglycerides; UACR: urinary albumin-to-creatinine ratio; WHR: waist-to-hip ratio

### Baseline characteristics of the study population

Age differed significantly across glycaemic groups, with higher mean age observed in participants with T2D compared with normoglycaemic and individuals with prediabetes. Glycaemic parameters, including fasting plasma glucose, postprandial glucose, and HbA1c, increased across groups, consistent with the glycaemic classification used to define these groups.

Markers of insulin resistance, particularly fasting insulin and HOMA-IR, were significantly higher in participants with diabetes. Anthropometric indices, including BMI, WHR, and BRI, also varied across groups. In addition, triglyceride levels, TG/HDL ratio, hs-CRP, and UACR differed significantly among glycaemic categories. Hypertension was present in 0%, 25.0%, and 69.2% of the normoglycaemic, prediabetes, and T2D groups, respectively (*p* < 0.001).

### Carotid plaque prevalence across glycaemic groups

**Fig. 1:**
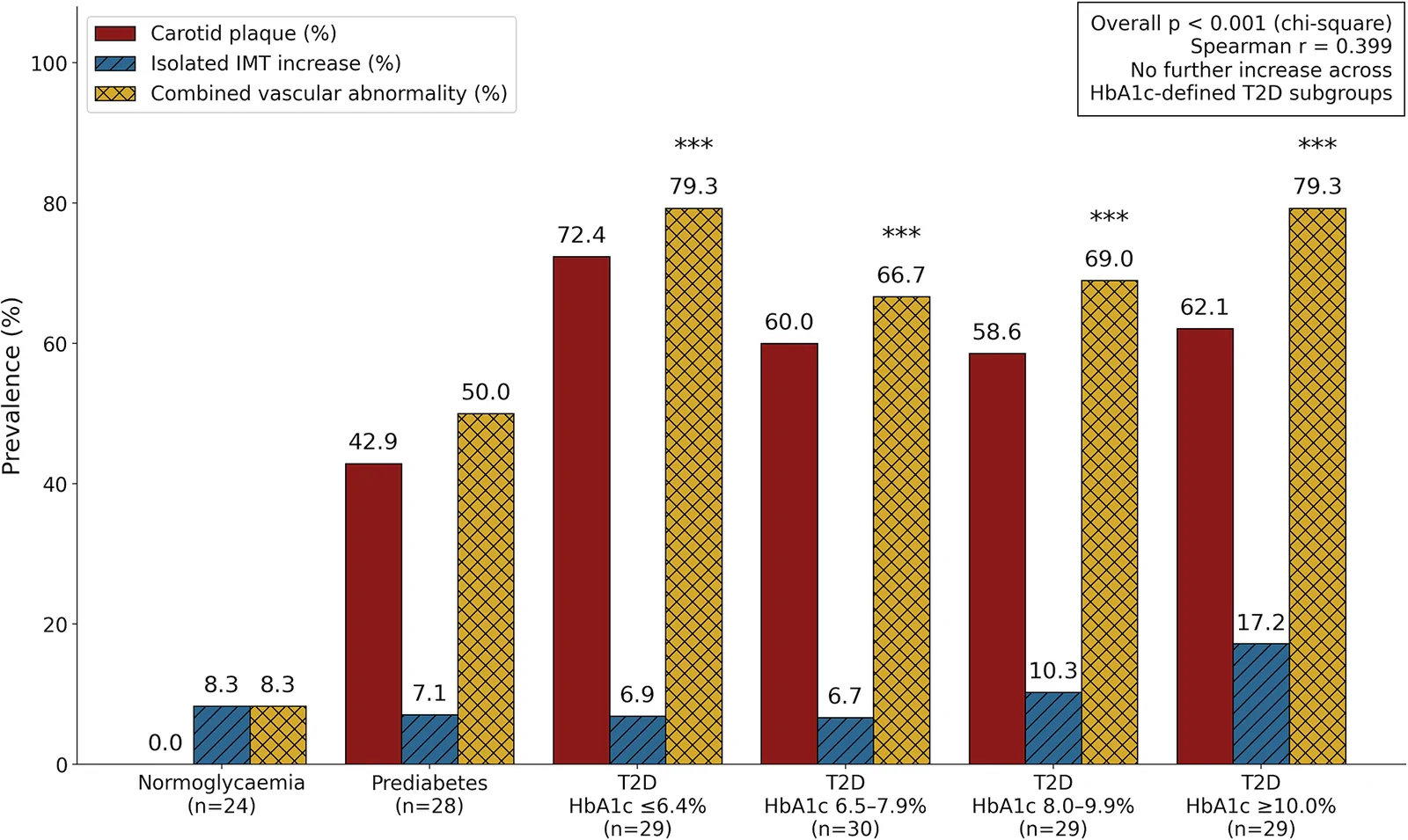
Prevalence of carotid plaque and subclinical vascular abnormality across the glycaemic spectrum. The prevalence of carotid plaque was markedly higher across the dysglycaemic spectrum than in normoglycaemia (p for trend < 0.001, Spearman *r* = 0.399). Half of individuals with prediabetes already exhibited subclinical vascular abnormalities — carotid plaque in 42.9% and isolated intima–media thickness (IMT) increase in a further 7.1% — highlighting the substantial burden of subclinical atherosclerosis already present at this early stage of dysglycaemia. In the normoglycaemic group, no carotid plaque was detected; isolated IMT increase was observed in 2 participants (8.3%). Carotid plaque was defined as a focal structure with echogenicity classified as Type I–IV according to the Gray–Weale classification. Isolated IMT increase was defined as increased IMT in the absence of overt plaque. *** *p* < 0.001 vs. normoglycaemia (Fisher’s exact test, pairwise comparison). Carotid plaque prevalence did not increase further across the HbA1c-defined T2D subgroups. IMT: intima–media thickness; T2D: type 2 diabetes mellitus

Carotid plaque prevalence differed significantly across glycaemic groups (*p* < 0.001). In normoglycaemic individuals, no carotid plaque was detected; however, isolated intima–media thickness increase was observed in 2 participants (8.3%), yielding a combined subclinical vascular abnormality prevalence of 8.3% in this group. In the prediabetes group, carotid plaque was present in 42.9% of participants; an additional 7.1% exhibited isolated intima–media thickness increase without overt plaque, yielding a combined subclinical vascular abnormality prevalence of 50.0%. Among patients with T2D, carotid plaque alone ranged from 58.6% to 72.4% across subgroups, while the combined outcome ranged from 66.7% to 79.3%. A significant overall association was observed between glycaemic category and combined vascular abnormality across the full spectrum (Spearman *r* = 0.399, *p* < 0.001), driven primarily by the marked difference between normoglycaemia and dysglycaemia; however, prevalence did not increase consistently across the HbA1c-defined T2D subgroups, indicating a plateau rather than a further increase within this range. The distribution of carotid plaque across glycaemic groups is presented in Table 2; Fig. 1.

**Table 2:** Distribution of carotid plaque, intima–media thickness increase, and combined subclinical vascular abnormality across glycaemic groups

Glycaemic group	*n*	Carotid plaque *n* (%)	Isolated IMT increase, *n* (%)	Combined vascular abnormality, *n* (%)	No vascular abnormality, *n* (%)
Normoglycaemia	24	0 (0.0)	2 (8.3)	2 (8.3)	22 (91.7)
Prediabetes	28	12 (42.9)	2 (7.1)	14 (50.0)	14 (50.0)
T2D (HbA1c ≤ 6.4%)	29	21 (72.4)	2 (6.9)	23 (79.3)	6 (20.7)
T2D (HbA1c 6.5–7.9%)	30	18 (60.0)	2 (6.7)	20 (66.7)	10 (33.3)
T2D (HbA1c 8.0–9.9%)	29	17 (58.6)	3 (10.3)	20 (69.0)	9 (31.0)
T2D (HbA1c ≥ 10.0%)	29	18 (62.1)	5 (17.2)	23 (79.3)	6 (20.7)

Carotid plaque was defined as a focal structure with echogenicity classified as Type I–IV according to the Gray–Weale classification. Isolated intima–media thickness increase was defined as intima–media thickness ≥ 1.0 mm in the absence of plaque. Combined vascular abnormality includes both carotid plaque and isolated IMT increase. p value for overall comparison across glycaemic groups was calculated using the chi-square test: *p* < 0.001. HbA1c: glycated haemoglobin; IMT: intima–media thickness; T2D: type 2 diabetes mellitus

### Carotid plaque morphology and stenosis severity

Carotid plaque morphology and stenosis severity were further evaluated among participants with detected plaques. According to the Gray–Weale classification, plaque morphology differed significantly across glycaemic groups (*p* < 0.001). Among participants with plaque, echolucent (Type I) plaques were proportionally more frequent in prediabetes (41.7% of plaque-positive participants, in whom Type I and Type IV plaques were equally represented), whereas echogenic (Type IV) plaques predominated in the T2D subgroups (61.1–72.2% of plaque-positive participants), with a correspondingly lower proportion of Type I plaques (5.6–22.2%) in these subgroups.

Carotid stenosis categories also differed across glycaemic groups when the whole cohort was considered (*p* < 0.001), primarily reflecting the higher plaque prevalence in dysglycaemia. However, when the analysis was restricted to participants with plaque, the proportion of plaques associated with stenosis did not differ significantly across the dysglycaemic groups (*p* = 0.113): 50.0% in prediabetes (6/12), 28.6% in the HbA1c ≤ 6.4% subgroup (6/21), 50.0% (9/18) and 52.9% (9/17) in the HbA1c 6.5–7.9% and 8.0–9.9% subgroups, respectively, and 72.2% (13/18) in the HbA1c ≥ 10.0% subgroup. A higher proportion of stenosis-associated plaques was therefore observed only in the subgroup with the highest HbA1c, and plaques detected in prediabetes were not more frequently non-stenotic than those detected in T2D. The distribution of plaque morphology and stenosis categories across glycaemic groups is summarised in Supplementary Table 2.

### Independent predictors of carotid atherosclerosis

Age, UACR, and total cholesterol were independently associated with carotid plaque presence after multivariable adjustment for cardiovascular risk factors, medication use, and macrovascular disease (Table 3; *n* = 169). Diabetes duration, HOMA-IR, and HbA1c did not retain independent predictive value after this adjustment. A sensitivity analysis excluding insulin-treated participants (*n* = 144) yielded consistent estimates for age (OR 1.06, 95% CI 1.00–1.12, *p* = 0.043) and total cholesterol (OR 1.76, 95% CI 1.15–2.69, *p* = 0.010), and HOMA-IR again showed no independent association (OR 0.95, 95% CI 0.85–1.07, *p* = 0.414); the association with UACR was attenuated to borderline significance in this smaller sample (OR 1.01, 95% CI 1.00–1.03, *p* = 0.059), which is most plausibly explained by the reduced sample size and by the exclusion of participants with the highest albuminuria (Supplementary Table 3). A further sensitivity analysis excluding macrovascular disease from the covariate set did not materially alter the findings (Supplementary Table 4). Detailed results of the multivariable analysis are presented in Table 3.

**Table 3:** Expanded multivariable logistic regression analysis for carotid plaque presence

Variable	Odds ratio (95% CI)	*p* value
Age (years)	1.07 (1.01–1.12)	0.014
Diabetes duration (months)	1.00 (0.99–1.01)	0.835
HOMA-IR	0.99 (0.92–1.07)	0.797
UACR (mg/g)	1.01 (1.00–1.02)	0.031
HbA1c (%)	1.01 (0.82–1.23)	0.925
Hypertension	1.17 (0.32–4.25)	0.810
Total cholesterol (mmol/L)	1.80 (1.21–2.67)	0.004
Statin use	1.35 (0.41–4.48)	0.623
RAAS blocker use	0.88 (0.25–3.13)	0.843
Antiplatelet use	1.97 (0.61–6.35)	0.257
Insulin use	1.12 (0.29–4.36)	0.867
eGFR (mL/min/1.73 m²)	0.99 (0.98–1.01)	0.417
Macrovascular disease	1.94 (0.82–4.62)	0.134
Oral antidiabetic use	1.35 (0.51–3.58)	0.553

Odds ratios are presented per unit increase. The expanded model included age, diabetes duration, HOMA-IR, UACR, HbA1c, hypertension, total cholesterol, statin use, RAAS blocker use, antiplatelet therapy, insulin therapy, eGFR, macrovascular disease, and oral antidiabetic therapy. Hypertension was defined as systolic blood pressure ≥ 140 mmHg, diastolic blood pressure ≥ 90 mmHg, or current use of antihypertensive medication. Macrovascular disease was defined as ischaemic heart disease and/or peripheral arterial disease (ascertained by an abnormal ankle–brachial index), excluding carotid atherosclerosis. The analysis included all 169 participants, none of whom had missing data for all covariates (model AUC = 0.821). CI: confidence interval; eGFR: estimated glomerular filtration rate; HbA1c: glycated haemoglobin; HOMA-IR: homeostasis model assessment of insulin resistance; RAAS: renin-angiotensin-aldosterone system; UACR: urinary albumin-to-creatinine ratio

### ROC analysis

**Fig. 2:**
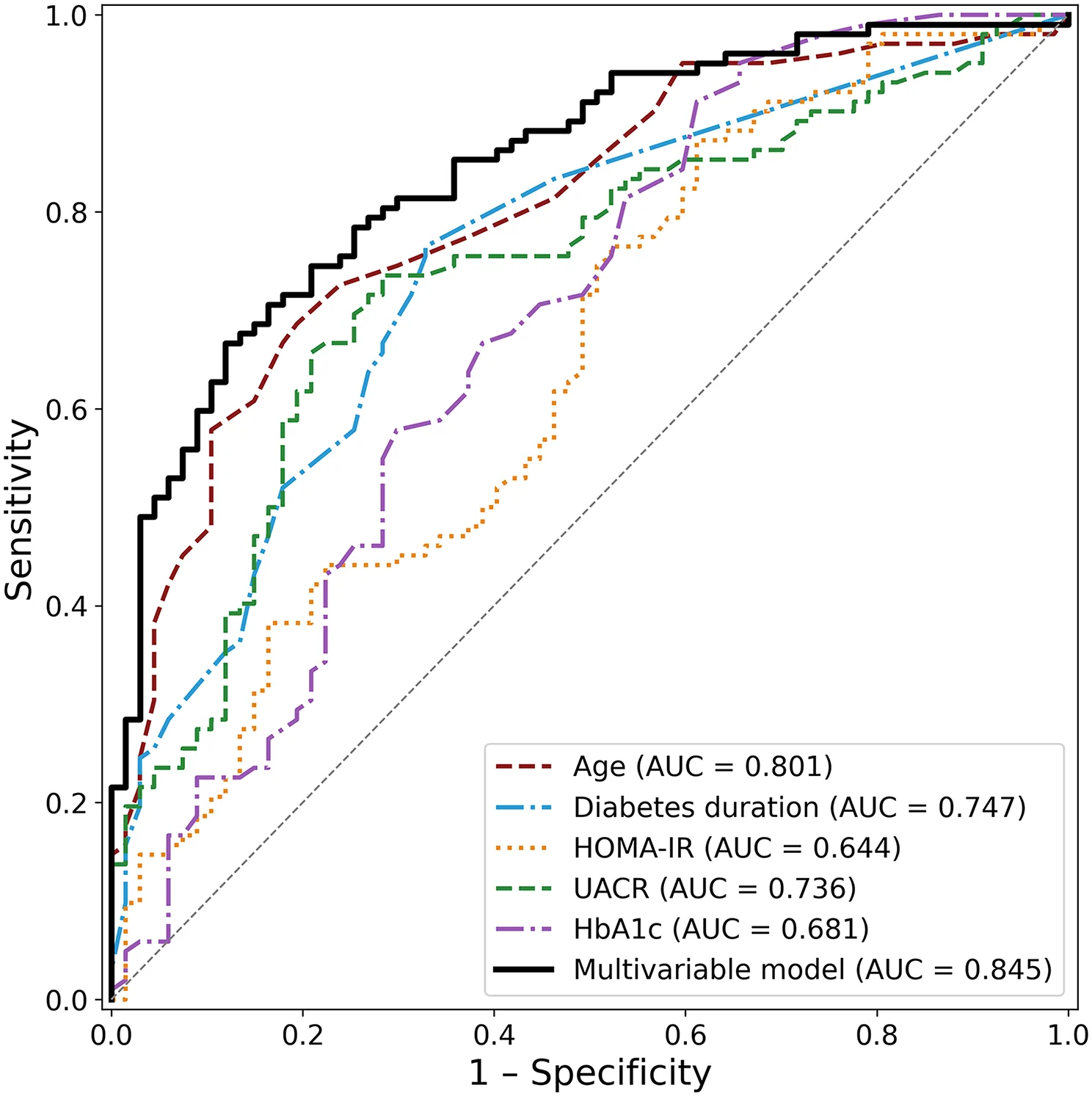
Receiver operating characteristic (ROC) curve analysis for the discrimination of subclinical vascular abnormality. ROC curves are shown for age, diabetes duration, UACR, HbA1c, HOMA-IR, and a multivariable model comprising these five variables. The outcome was the combined outcome of carotid plaque or isolated intima–media thickness increase, as specified for ROC analysis. The five-variable multivariable model demonstrated good discriminatory performance (AUC 0.845), followed by age (AUC 0.801), diabetes duration (AUC 0.747), UACR (AUC 0.736), HbA1c (AUC 0.681), and HOMA-IR (AUC 0.644). All analyses, including the multivariable model, were performed in all 169 participants. AUC values with 95% confidence intervals are presented in Table 4. AUC: area under the receiver operating characteristic curve; HbA1c: glycated haemoglobin; HOMA-IR: homeostasis model assessment of insulin resistance; UACR: urinary albumin-to-creatinine ratio

ROC curve analysis was performed to evaluate the discriminatory ability of selected clinical and metabolic variables for the combined outcome of carotid plaque or isolated intima–media thickness increase. Age demonstrated the highest discriminatory performance (AUC = 0.801, 95% CI 0.734–0.864), followed by diabetes duration (AUC = 0.747), UACR (AUC = 0.736), HbA1c (AUC = 0.681), and HOMA-IR (AUC = 0.644). The optimal cut-off value for age was 54 years (sensitivity 68.6%, specificity 80.6%). A UACR threshold of 8.9 mg/g, which is still within the conventional normal range, was associated with vascular abnormality in this exploratory ROC analysis (sensitivity 73.5%, specificity 71.6%). A multivariable model comprising age, diabetes duration, HOMA-IR, UACR, and HbA1c showed good discrimination for this combined outcome (AUC = 0.845, 95% CI 0.787–0.900; *n* = 169), whereas the expanded multivariable model for carotid plaque presence (Table 3) yielded an AUC of 0.821. The ROC curves for the principal predictors are presented in Fig. 2; Table 4.

**Table 4:** Receiver operating characteristic curve analysis for discrimination of carotid plaque or isolated intima–media thickness increase

Variable	AUC (95% CI)	Optimal cut-off (Youden)	Sensitivity (%)	Specificity (%)	*n*
Age (years)	0.801 (0.734–0.864)	54.0 years	68.6	80.6	169
Diabetes duration (months)	0.747 (0.669–0.817)	6.0 months	76.5	67.2	169
HOMA-IR	0.644 (0.554–0.728)	2.2	87.3	38.8	169
UACR (mg/g)	0.736 (0.657–0.812)	8.9 mg/g	73.5	71.6	169
HbA1c (%)	0.681 (0.592–0.763)	5.6%	91.2	38.8	169
Total cholesterol (mmol/L)	0.614 (0.526–0.698)	4.76 mmol/L	64.7	56.7	169
Multivariable model (5 variables)	0.845 (0.787–0.900)	—	66.7	88.1	169

AUC values were calculated using the non-parametric (Mann–Whitney) method; 95% confidence intervals were derived by bootstrap resampling (2000 iterations). Optimal cut-off values were determined using the Youden index (sensitivity + specificity − 1). Univariable analyses included all participants with available data for the variable concerned; the multivariable model comprised age, diabetes duration, HOMA-IR, UACR and HbA1c and was fitted in all 169 participants, none of whom had missing data for these variables. All p values < 0.001 except total cholesterol (*p* = 0.012). AUC: area under the receiver operating characteristic curve; CI: confidence interval; HbA1c: glycated haemoglobin; HOMA-IR: homeostasis model assessment of insulin resistance; UACR: urinary albumin-to-creatinine ratio

## Discussion

In the present study, carotid atherosclerosis was markedly more prevalent across the dysglycaemic spectrum than in normoglycaemia, extending from prediabetes to established type 2 diabetes. A particularly notable finding was the high prevalence of carotid plaque among individuals with prediabetes, indicating that structural vascular alterations may be present well before the clinical diagnosis of diabetes. In the normoglycaemic group, no carotid plaque was detected; however, isolated intima–media thickness increase was observed in 2 participants (8.3%). The absence of overt plaque likely reflects the younger age and smaller sample size of this subgroup. In addition to glycaemic status, age, UACR, and total cholesterol were independently associated with carotid plaque presence after adjustment for cardiovascular risk factors, medication use, and macrovascular disease, whereas diabetes duration, HOMA-IR, and HbA1c alone were not independent predictors.

Unlike many previous studies focusing primarily on carotid intima–media thickness, the present study comprehensively evaluated carotid plaque presence, morphology, and stenosis across the entire glycaemic spectrum [21, 22].

Prediabetes is characterised by insulin resistance, β-cell dysfunction, chronic low-grade inflammation, and endothelial dysfunction, all of which may contribute to early vascular damage [6, 7]. Beyond these mechanisms, small dense LDL particles have been shown to predict subsequent intima–media thickness progression, while platelet hyperreactivity accompanied by a pro-inflammatory profile has also been associated with early vascular damage in prediabetes [8, 9].

Carotid ultrasonography is widely used for the detection of subclinical atherosclerosis, and accumulating evidence suggests that carotid plaque provides stronger prognostic information than intima–media thickness alone regarding future cardiovascular events [3]. Contemporary imaging approaches increasingly emphasise plaque morphology and stenosis severity in addition to plaque presence when evaluating atherosclerotic burden [4, 5]. Beyond plaque prevalence, plaque morphology also differed across glycaemic categories in the present study, with echolucent plaques proportionally more common in prediabetes and echogenic plaques predominating in T2D; although the cross-sectional design precludes inference regarding temporal progression, this observation suggests that plaque phenotype across the dysglycaemic spectrum merits further investigation. Among plaque-positive participants, however, the proportion of stenosis-associated plaques did not differ significantly across the dysglycaemic categories and was higher only in the subgroup with HbA1c ≥ 10.0%, suggesting that stenosis severity tracks markedly elevated glycaemia rather than dysglycaemia per se.

Another important observation is that markers reflecting cumulative metabolic exposure appeared more strongly associated with carotid plaque than glycaemic control alone: age, UACR, and total cholesterol remained independently associated with plaque presence in the expanded multivariable model, whereas diabetes duration and HOMA-IR showed discriminatory value in univariable ROC analysis but did not retain independent predictive value after multivariable adjustment. Notably, although HbA1c demonstrated moderate discriminatory ability in ROC analysis (AUC 0.681), it did not retain independent predictive value after adjustment for age, UACR, total cholesterol, and other cardiovascular risk factors and comorbidities in the expanded multivariable model — suggesting that its univariate association with carotid plaque is largely mediated by these cumulative cardiometabolic factors [23–25]. Dynamic measures of glucose metabolism, such as 2-hour post-challenge glucose, have been shown to be more strongly associated with subclinical atherosclerosis than HbA1c, suggesting that static glycaemic markers may underestimate vascular risk in dysglycaemia [26, 27]. Consistent with these observations, a recent predictive model for carotid atherosclerosis in 1,258 patients with T2D identified age, blood pressure, lipid markers, and renal function as independent predictors, whereas HbA1c was not retained in the final multivariable model [28].

These results highlight the multifactorial nature of atherosclerosis in dysglycaemia, supporting a cardiometabolic risk framework in which cumulative burden — reflected by age, UACR, and total cholesterol — contributes to vascular injury independently of glycaemic control, consistent with the concept that HbA1c alone is an insufficient surrogate for overall cardiovascular risk in dysglycaemia [7]. UACR, in particular, is widely recognised as a marker of systemic endothelial dysfunction and generalised vascular damage [29]. Although HOMA-IR was not independently associated with carotid plaque in the present adjusted model, insulin resistance has been proposed to promote endothelial injury and albuminuria through overlapping cardiorenal pathways [7].

eGFR was similarly incorporated into the expanded multivariable model but was not independently associated with carotid plaque, in contrast to Di Marco et al. [30], who reported increased carotid plaque burden specifically in the non-albuminuric diabetic kidney disease phenotype, linked to inflammatory pathways; this suggests that UACR may capture renal-related vascular risk more directly than eGFR in the present population. These findings should also be interpreted within the broader cardiometabolic context: Di Pino et al. [31] reported that metabolic syndrome contributes to early vascular injury independently of altered glucose tolerance, reinforcing that vascular injury across the glycaemic spectrum likely reflects multiple interacting cardiometabolic factors rather than glycaemic status alone; metabolic syndrome itself was not evaluated as a separate construct in the present study.

From a clinical perspective, the high prevalence of carotid plaque observed in individuals with prediabetes carries important implications for cardiovascular risk stratification, as current prevention strategies mainly focus on individuals with established diabetes or overt cardiovascular disease, whereas the present data indicate that structural vascular alterations may already be present during earlier stages of dysglycaemia. Given that half of individuals with prediabetes in this cohort exhibited subclinical vascular abnormalities, this approach may merit evaluation in future prospective studies [32]. Non-invasive vascular imaging methods such as carotid ultrasonography may therefore provide additional information for identifying individuals at increased cardiovascular risk beyond traditional metabolic markers [33]. Based on the present findings, carotid ultrasonography may be particularly informative in individuals with prediabetes accompanied by elevated UACR, prolonged dysglycaemia, or advanced age, as these factors were most strongly associated with subclinical atherosclerosis in the present cohort [24, 33].

Several limitations should be considered when interpreting these findings. First, the cross-sectional design does not allow causal inference regarding the relationship between glycaemic status and carotid atherosclerosis; accordingly, reverse causality cannot be excluded, and the temporal onset of the observed vascular changes cannot be determined. Second, the relatively modest sample size and single-center design may limit the generalizability of the findings. Third, although plaque morphology was assessed using established ultrasonographic criteria, more advanced imaging techniques may provide additional insights into plaque composition and vulnerability; all examinations were performed and interpreted by a single experienced radiologist, and formal intraobserver reproducibility testing was not performed as part of the original study protocol. Because stenosis grading in this retrospective cohort relied on integrated clinical assessment rather than a single documented velocity threshold, exact reproducibility of stenosis percentage assignment cannot be verified; however, this approach is consistent with international consensus recommending morphological assessment as the primary criterion for low-to-moderate degrees of stenosis [20], and plaque presence—the primary study outcome—was determined independently of precise stenosis grading. Fourth, because the normoglycaemic control group was ascertained from outpatient clinic attendees seeking routine health evaluation rather than a separately recruited community-based sample, some degree of selection bias inherent to hospital-based control recruitment cannot be excluded. Another limitation is the difference in mean age across glycaemic groups, with normoglycaemic controls being younger than participants with prediabetes and T2D. However, age was included as a covariate in the multivariable model and remained an independent predictor of carotid plaque, suggesting that the association observed for UACR is not solely explained by age differences. In particular, the absence of carotid plaque in the normoglycaemic group should be interpreted with caution, as this subgroup was both smaller and younger; the observed finding may reflect sampling variability and age-related differences in plaque accrual rather than a true biological absence of atherosclerosis in normoglycaemia. A further limitation concerns the macrovascular disease covariate included in the expanded model. In the original cohort, peripheral arterial disease was ascertained by an abnormal ankle–brachial index rather than by a documented clinical event, so this variable largely captures screen-detected subclinical peripheral atherosclerosis; adjusting for it may therefore constitute over-adjustment for a marker of the same underlying disease process. Reassuringly, excluding this covariate did not materially alter the findings (Supplementary Table 4). In addition, with 86 events and 14 covariates the events-per-variable ratio in the expanded model was approximately 6, below the conventionally recommended threshold of 10, and the individual coefficient estimates should accordingly be interpreted as adjusted associations rather than as precise effect sizes. Finally, a small number of biologically implausible laboratory and anthropometric values identified during data verification were treated as missing; although these affected fewer than 1% of observations and none of the primary model covariates, they illustrate the constraints inherent to the retrospective use of routinely collected clinical data.

Despite these limitations, the study also has several strengths. Participants across the entire glycaemic spectrum were included, enabling comprehensive evaluation of carotid atherosclerosis from normoglycaemia to advanced T2D. Furthermore, the combined assessment of plaque presence, morphology, and stenosis severity provided a detailed characterisation of carotid atherosclerotic burden. Adjustment for major cardiovascular risk factors, medication use, and macrovascular disease did not materially alter the principal findings, and the results were confirmed in two prespecified sensitivity analyses, supporting the robustness of the observed associations.

## Conclusions

Carotid plaque prevalence was markedly higher across the dysglycaemic spectrum than in normoglycaemia and was already substantial among individuals with prediabetes. Age, UACR, and total cholesterol were independent predictors of carotid atherosclerosis, whereas diabetes duration, HOMA-IR, and HbA1c were not independently associated after multivariable adjustment. These findings highlight the importance of cardiometabolic factors beyond glycaemic control alone in determining carotid atherosclerotic burden across the glycaemic spectrum. Carotid ultrasonography, including assessment of plaque morphology, may provide additional information for cardiovascular risk stratification, particularly in individuals with prediabetes. This potential role merits evaluation in future prospective studies.

## Supplementary Information

Below is the link to the electronic supplementary material.

Supplementary Material 1

## Data Availability

The datasets used and/or analysed during the current study are available from the corresponding author on reasonable request.

## Abbreviations

ABI: Ankle–brachial index
ADA: American Diabetes Association
AUC: Area under the receiver operating characteristic curve
BMI: Body mass index
BRI: Body Roundness Index
CKD-EPI: Chronic Kidney Disease Epidemiology Collaboration
DBP: Diastolic blood pressure
ECST: European Carotid Surgery Trial
EDV: End-diastolic velocity
eGFR: Estimated glomerular filtration rate
FPG: Fasting plasma glucose
HDL-C: High-density lipoprotein cholesterol
HOMA-IR: Homeostasis model assessment of insulin resistance
hs-CRP: High-sensitivity C-reactive protein
HbA1c: Glycated haemoglobin
ICA/CCA: Internal carotid artery-to-common carotid artery
IMT: Intima–media thickness
IQR: Interquartile range
LDL-C: Low-density lipoprotein cholesterol
NGSP: National Glycohemoglobin Standardization Program
OGTT: Oral glucose tolerance test
PPG: Postprandial glucose
PSV: Peak systolic velocity
RAAS: Renin-angiotensin-aldosterone system
ROC: Receiver operating characteristic
SBP: Systolic blood pressure
T2D: Type 2 diabetes mellitus
TG: Triglycerides
TG/HDL: Triglyceride-to-HDL cholesterol ratio
UACR: Urinary albumin-to-creatinine ratio
WHR: Waist-to-hip ratio
