# Supplementary material for: Carotid plaque burden across the glycaemic spectrum: a cross-sectional study
Source: BMC Endocr Disord. 2026 Aug 1;26:269. doi: 10.1186/s12902-026-02455-z (PMC13563818; doi:10.1186/s12902-026-02455-z)
Supplement: Supplementary file 1 [file 12902_2026_2455_MOESM1_ESM.docx]

**Supplementary Table 1. Baseline characteristics of the study population according to glycaemic subgroup**

| **Variable** | **Normoglycaemia (n=24)** | **Prediabetes (n=28)** | **T2D HbA1c ≤6.4% (n=29)** | **T2D HbA1c 6.5–7.9% (n=30)** | **T2D HbA1c 8.0–9.9% (n=29)** | **T2D HbA1c ≥10.0% (n=29)** | **p value** |
| --- | --- | --- | --- | --- | --- | --- | --- |
| Age (years) | 47.96 ± 5.75 | 52.14 ± 8.64 | 58.14 ± 9.22 | 56.23 ± 9.56 | 56.03 ± 7.61 | 57.14 ± 8.90 | <0.001 |
| Male sex, n (%) | 13 (54.2) | 15 (53.6) | 15 (51.7) | 16 (53.3) | 14 (48.3) | 15 (51.7) | 0.998 |
| BMI (kg/m²) | 27.25 ± 3.11 | 31.79 ± 5.70 | 32.83 ± 5.48 | 33.83 ± 5.52 | 34.83 ± 5.26 | 35.66 ± 5.86 | <0.001 |
| WHR | 0.91 ± 0.06 | 0.98 ± 0.07 | 0.97 ± 0.06 | 0.98 ± 0.06 | 1.00 ± 0.05 | 1.00 ± 0.05 | <0.001 |
| BRI | 5.07 ± 1.17 | 7.17 ± 2.25 | 7.66 ± 2.63 | 7.86 ± 2.32 | 8.40 ± 2.02 | 8.77 ± 2.54 | <0.001 |
| Diabetes duration (months) | — | — | 48.0 (12.0–120.0) | 84.0 (51.0–120.0) | 96.0 (24.0–156.0) | 96.0 (60.0–180.0) | 0.072 |
| FPG (mmol/L) | 4.68 ± 0.30 | 5.74 ± 0.49 | 6.45 ± 0.97 | 7.70 ± 0.99 | 10.14 ± 2.23 | 14.70 ± 3.02 | <0.001 |
| PPG (mmol/L) | 4.94 ± 0.58 | 6.85 ± 1.64 | 7.52 ± 2.40 | 10.99 ± 2.46 | 14.30 ± 3.32 | 19.40 ± 4.04 | <0.001 |
| HbA1c (%) | 5.10 ± 0.18 | 5.74 ± 0.24 | 5.88 ± 0.35 | 7.01 ± 0.46 | 8.87 ± 0.65 | 11.32 ± 1.14 | <0.001 |
| Fasting insulin (µU/mL) | 7.99 ± 4.36 | 14.29 ± 7.12 | 15.07 ± 8.18 | 17.91 ± 10.55 | 16.11 ± 14.68 | 13.92 ± 8.43 | <0.001 |
| HOMA-IR | 1.4 (1.2–1.9) | 3.4 (2.2–4.4) | 3.5 (2.8–5.3) | 6.0 (3.1–7.8) | 5.1 (3.1–7.7) | 7.4 (6.1–10.2) | <0.001 |
| Total cholesterol (mmol/L) | 4.58 ± 1.07 | 4.94 ± 0.96 | 5.04 ± 1.03 | 5.50 ± 1.38 | 5.01 ± 1.11 | 4.98 ± 0.90 | 0.229 |
| LDL-C (mmol/L) | 2.3 (1.8–3.1) | 2.9 (2.1–3.3) | 3.1 (2.5–3.5) | 3.0 (2.8–3.5) | 3.0 (2.0–3.3) | 2.9 (2.5–3.2) | 0.068 |
| HDL-C (mmol/L) | 1.32 ± 0.36 | 1.10 ± 0.25 | 1.11 ± 0.20 | 1.12 ± 0.25 | 1.06 ± 0.27 | 1.01 ± 0.26 | 0.016 |
| TG (mmol/L) | 1.2 (0.8–1.7) | 1.9 (1.2–2.3) | 1.6 (1.3–2.3) | 2.0 (1.5–2.9) | 2.1 (1.5–3.3) | 2.2 (1.7–3.1) | <0.001 |
| hs-CRP (mg/L) | 1.2 (0.8–1.7) | 2.5 (1.4–3.4) | 2.5 (1.5–3.4) | 2.6 (2.5–4.1) | 3.8 (2.5–7.4) | 4.5 (2.5–9.2) | <0.001 |
| UACR (mg/g) | 5.0 (3.9–6.2) | 7.0 (4.0–8.6) | 9.3 (5.3–24.4) | 17.6 (8.9–62.3) | 14.4 (7.7–74.1) | 21.1 (16.0–34.8) | <0.001 |
| eGFR (mL/min/1.73m²) | 104.87 ± 18.42 | 94.99 ± 20.51 | 105.00 ± 21.11 | 102.55 ± 23.24 | 107.41 ± 23.77 | 110.33 ± 29.40 | 0.426 |
| Hypertension, n (%) | 0 (0.0) | 7 (25.0) | 23 (79.3) | 19 (63.3) | 17 (58.6) | 22 (75.9) | <0.001 |
| Macrovascular disease, n (%) | 0 (0.0) | 19 (67.9) | 25 (86.2) | 26 (86.7) | 23 (79.3) | 22 (75.9) | <0.001 |
| Smoking, n (%) | 3 (12.5) | 8 (28.6) | 4 (13.8) | 7 (23.3) | 8 (27.6) | 6 (20.7) | 0.589 |

Data are mean ± SD or median (IQR) for continuous variables and n (%) for categorical variables. p values were derived using the Kruskal–Wallis test (continuous) or chi-square test (categorical) across all six subgroups. Diabetes duration is not applicable (—) for the normoglycaemia and prediabetes groups. BRI: Body Roundness Index; other abbreviations as in Table 1.

**Supplementary Table 2. Distribution of carotid plaque morphology and stenosis severity across glycaemic groups**

| **Variable** | **Normoglycaemia (n=24)** | **Prediabetes (n=28)** | **T2D HbA1c ≤6.4% (n=29)** | **T2D HbA1c 6.5–7.9% (n=30)** | **T2D HbA1c 8.0–9.9% (n=29)** | **T2D HbA1c ≥10.0% (n=29)** |
| --- | --- | --- | --- | --- | --- | --- |
| Type I (uniformly echolucent) | 0 (0.0) | 5 (17.9) | 4 (13.8) | 1 (3.3) | 1 (3.4) | 4 (13.8) |
| Type II (predominantly echolucent) | 0 (0.0) | 2 (7.1) | 0 (0.0) | 3 (10.0) | 4 (13.8) | 2 (6.9) |
| Type III (predominantly echogenic) | 0 (0.0) | 0 (0.0) | 2 (6.9) | 1 (3.3) | 0 (0.0) | 1 (3.4) |
| Type IV (uniformly echogenic) | 0 (0.0) | 5 (17.9) | 15 (51.7) | 13 (43.3) | 12 (41.4) | 11 (37.9) |
| Normal Doppler (no plaque) | 22 (91.7) | 14 (50.0) | 6 (20.7) | 10 (33.3) | 9 (31.0) | 6 (20.7) |
| Isolated IMT increase (no plaque) | 2 (8.3) | 2 (7.1) | 2 (6.9) | 2 (6.7) | 3 (10.3) | 5 (17.2) |
| No plaque, no stenosis | 24 (100.0) | 16 (57.1) | 8 (27.6) | 12 (40.0) | 12 (41.4) | 11 (37.9) |
| Plaque present, no stenosis | 0 (0.0) | 6 (21.4) | 15 (51.7) | 9 (30.0) | 8 (27.6) | 5 (17.2) |
| Plaque present, with stenosis | 0 (0.0) | 6 (21.4) | 6 (20.7) | 9 (30.0) | 9 (31.0) | 13 (44.8) |
| Stenotic plaques / all plaques, n (%) | — | 6/12 (50.0) | 6/21 (28.6) | 9/18 (50.0) | 9/17 (52.9) | 13/18 (72.2) |

Data are n (percentage of the total group n, including participants without plaque), except for the final row, which is expressed as a percentage of plaque-positive participants. Plaque morphology was classified according to the Gray–Weale classification. The distributions of plaque morphology and of stenosis category across the six groups differed significantly (chi-square, p < 0.001 for both). However, when restricted to plaque-positive participants, the proportion of stenosis-associated plaques did not differ significantly across the five dysglycaemic groups (chi-square, p = 0.113). IMT: intima–media thickness; T2D: type 2 diabetes mellitus.

**Supplementary Table 3. Expanded multivariable logistic regression for carotid plaque presence after exclusion of insulin-treated participants**

| **Variable** | **Odds ratio (95% CI)** | **p value** |
| --- | --- | --- |
| Age (years) | 1.06 (1.00–1.12) | 0.043 |
| Diabetes duration (months) | 1.01 (1.00–1.02) | 0.180 |
| HOMA-IR | 0.95 (0.85–1.07) | 0.414 |
| UACR (mg/g) | 1.01 (1.00–1.03) | 0.059 |
| HbA1c (%) | 0.97 (0.77–1.22) | 0.795 |
| Hypertension | 0.88 (0.21–3.73) | 0.864 |
| Total cholesterol (mmol/L) | 1.76 (1.15–2.69) | 0.010 |
| Statin use | 2.01 (0.42–9.51) | 0.380 |
| RAAS blocker use | 1.66 (0.38–7.19) | 0.499 |
| Antiplatelet use | 1.12 (0.26–4.80) | 0.877 |
| eGFR (mL/min/1.73m²) | 0.99 (0.97–1.01) | 0.290 |
| Macrovascular disease | 2.31 (0.86–6.19) | 0.096 |
| Oral antidiabetic use | 0.84 (0.26–2.71) | 0.775 |

Sensitivity analysis performed after excluding all insulin-treated participants (n = 25) from the cohort (n = 169), leaving 144 participants, all with complete data for the model covariates; of these, 67 had carotid plaque. The insulin therapy covariate was necessarily omitted. Model AUC = 0.825. Abbreviations as in Table 3.

**Supplementary Table 4. Expanded multivariable logistic regression for carotid plaque presence after exclusion of the macrovascular disease covariate**

| **Variable** | **Odds ratio (95% CI)** | **p value** |
| --- | --- | --- |
| Age (years) | 1.07 (1.02–1.13) | 0.007 |
| Diabetes duration (months) | 1.00 (0.99–1.01) | 0.956 |
| HOMA-IR | 1.00 (0.93–1.08) | 0.966 |
| UACR (mg/g) | 1.01 (1.00–1.02) | 0.032 |
| HbA1c (%) | 1.02 (0.83–1.24) | 0.882 |
| Hypertension | 1.40 (0.40–4.91) | 0.602 |
| Total cholesterol (mmol/L) | 1.77 (1.20–2.60) | 0.004 |
| Statin use | 1.26 (0.38–4.15) | 0.707 |
| RAAS blocker use | 0.84 (0.24–2.97) | 0.790 |
| Antiplatelet use | 1.96 (0.61–6.26) | 0.256 |
| Insulin use | 1.09 (0.28–4.20) | 0.906 |
| eGFR (mL/min/1.73m²) | 0.99 (0.97–1.01) | 0.424 |
| Oral antidiabetic use | 1.51 (0.58–3.95) | 0.403 |

Sensitivity analysis performed after removing macrovascular disease from the covariate set, because peripheral arterial disease in the original cohort was ascertained by an abnormal ankle–brachial index rather than by a documented clinical event, and adjusting for it may therefore represent over-adjustment for a marker of the same underlying atherosclerotic process. The analysis was performed in all 169 participants, none of whom had missing data for these covariates (model AUC = 0.819). Age, UACR and total cholesterol remained independently associated with carotid plaque, confirming that the principal findings are not dependent on this covariate. Abbreviations as in Table 3.
